# Supplementary figures and images for: Combining CDK4/6 inhibitors ribociclib and palbociclib with cytotoxic agents does not enhance cytotoxicity
Source: PLoS One. 2019 Oct 10;14(10):e0223555. doi: 10.1371/journal.pone.0223555 (PMC6786609; doi:10.1371/journal.pone.0223555)

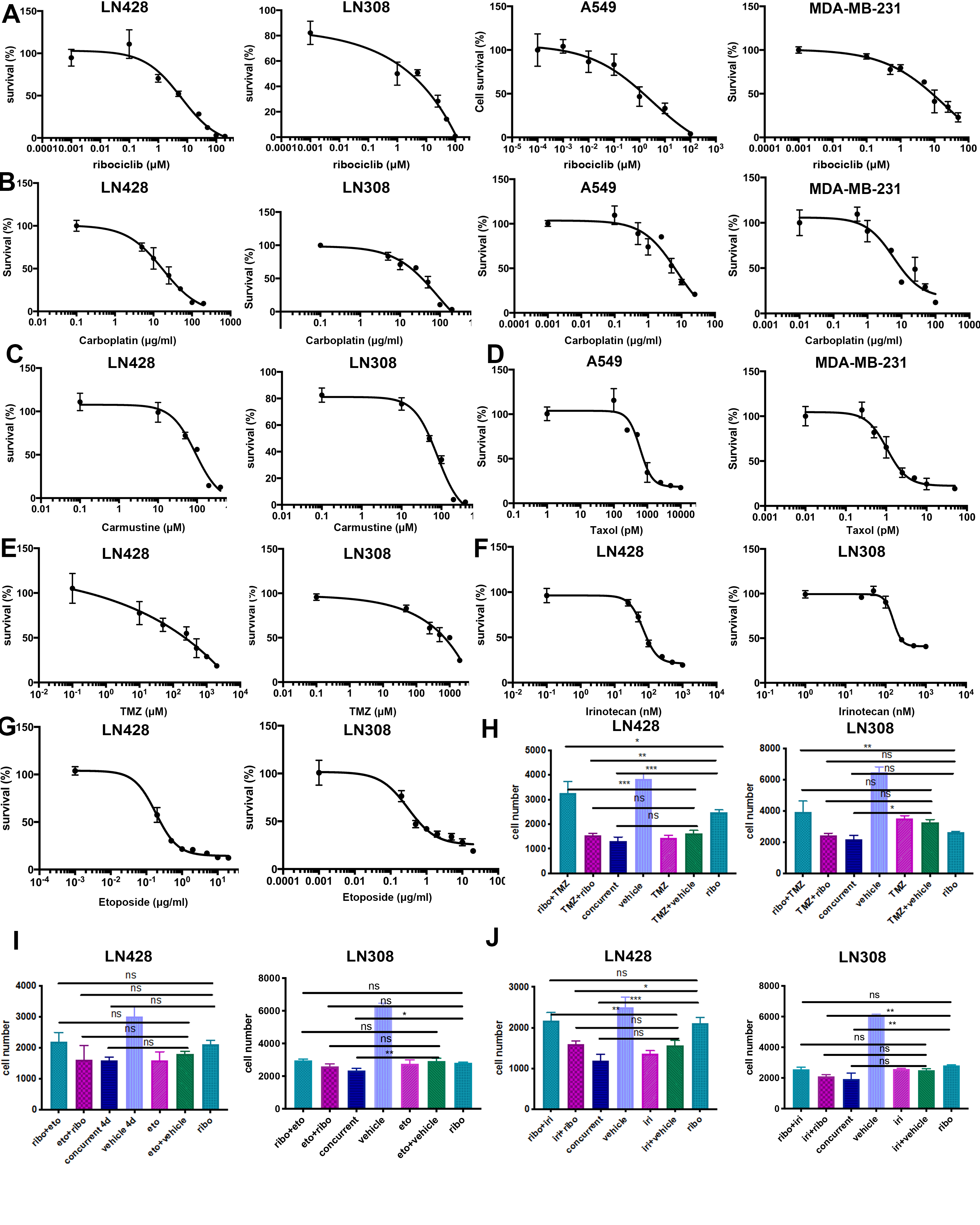

Supplement: S1 Fig — (A-G) Representative dose-response curves of 3 independent biological repeats of ribociclib (A), carboplatin (B), carmustine (C), paclitaxel (Taxol) (D), temozolomide (TMZ) (E), etoposide (F) and irinotecan (G) for the growth inhibition of LN428, LN308, A549 and MDA-MB-231 are shown. Each data point was done in triplicates. (H-J) Graphs of representative cytotoxicity assay of 3 independent repeats of the various combinations of ribociclib as shown in Fig 1B at the IC50 concentration for each drug in LN428 and LN308 cells with TMX (H), etoposide (I) and irinotecan (J). ribo: ribociclib; TMZ: temozolomide; eto: etoposide; iri: irinotecan. All values are numbers of live cells remaining in culture at the end of treatment and presented as mean (SD). P-value was calculated by one way ANOVA: *, p<0.033; **, p <0.02; ***, p < 0.001. (TIF) [file pone.0223555.s001.tif]

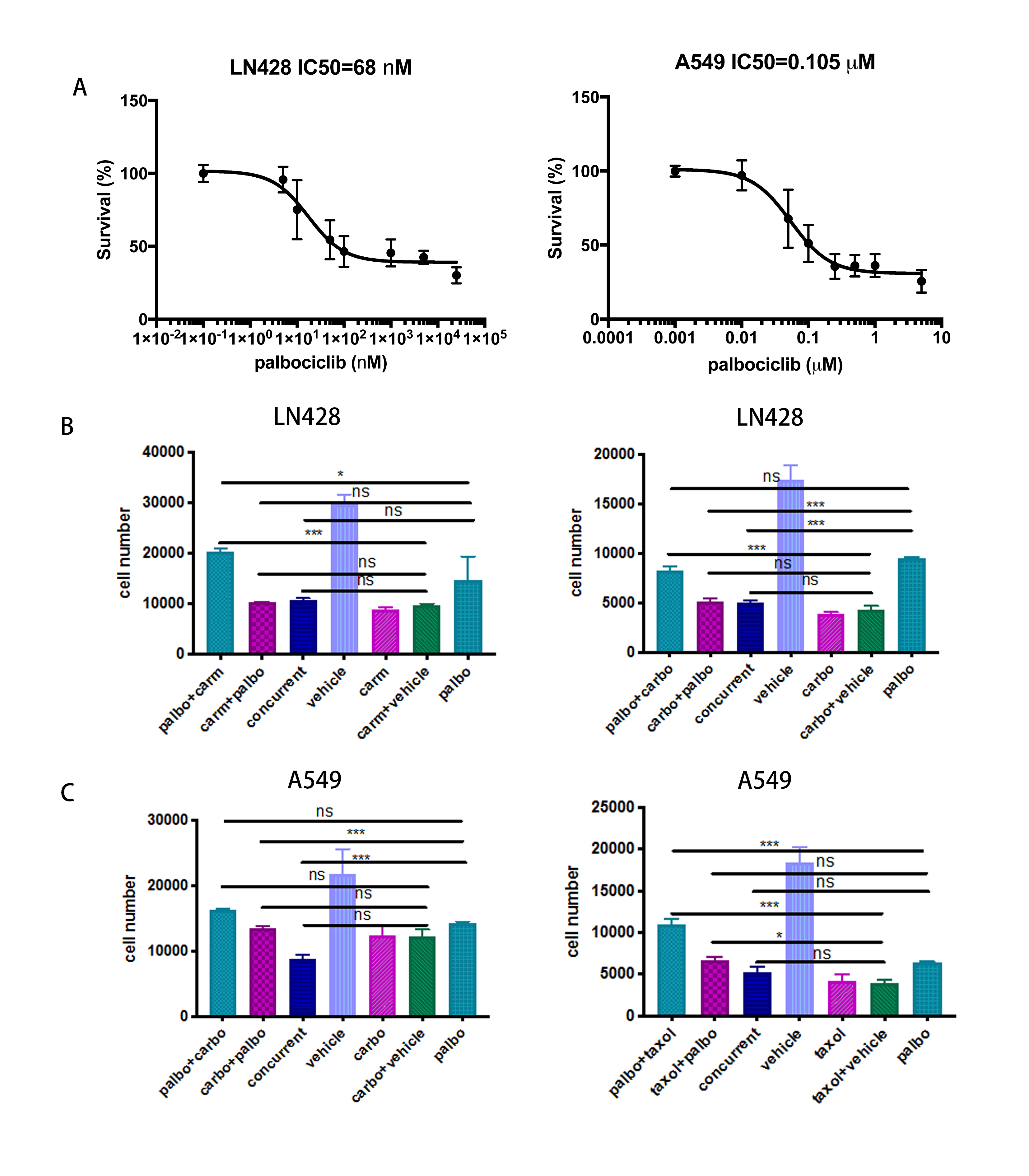

Supplement: S2 Fig — (A) Representative dose-response curves of 3 independent biological repeats of palbociclib in LN428 and A549 cells are shown. Each data point was done in triplicates. (B-C) Graphs of representative cytotoxicity assay of 3 independent repeats of the various combinations of palbociclib at its IC50 concentration in LN428 (B) and A549 (C) cells with indicated cytotoxic drugs. palbo: palbociclib; carm: carmustine; carbo: carboplatin. All values are numbers of live cells remaining in culture at the end of treatment and presented as mean (SD). P-value was calculated by one way ANOVA: *, p<0.033; **, p <0.02; ***, p < 0.001. (TIF) [file pone.0223555.s002.tif]

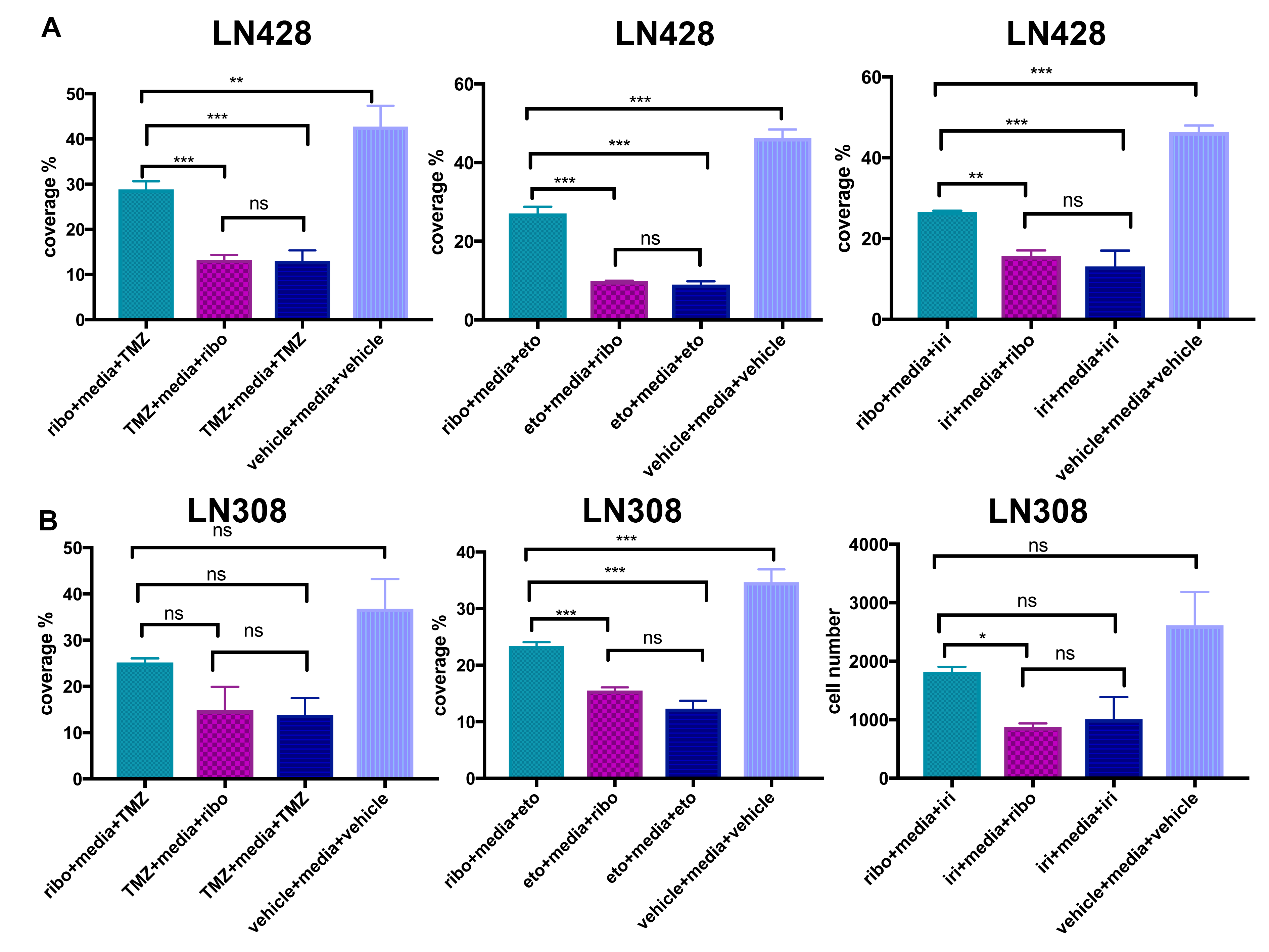

Supplement: S3 Fig — (A-B) Graphs of representative cytotoxicity assay of 3 independent repeats of the various combinations of ribociclib and indicated cytotoxic drugs as shown in Fig 2A at the IC50 concentration for each drug in LN428 (A) and LN308 (B) cells (E). All values are numbers of live cells remaining in culture at the end of treatment and presented as mean (SD). P-value was calculated by one way ANOVA: *, p<0.033; **, p <0.02; ***, p < 0.001. (TIF) [file pone.0223555.s003.tif]

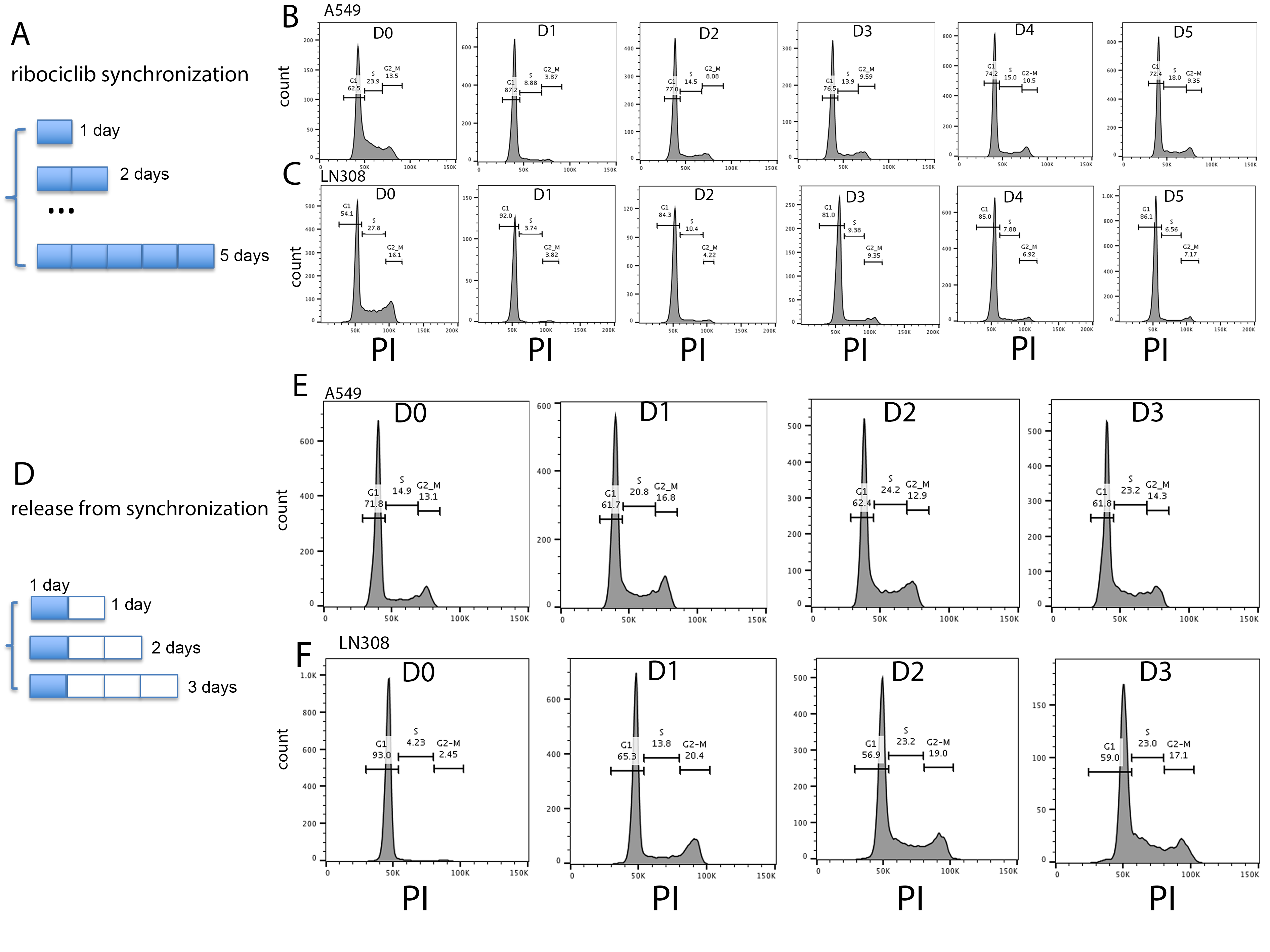

Supplement: S4 Fig — (A) A diagram of G1/S synchronization by ribociclib. (B-C). Representative histograms of cell cycle analysis of A549 (B) and LN308 (C) cancer cell lines treated with ribociclib for 0–5 days (D0-D5). Percentages of cells at different stages of the cell cycle are listed. (D) A diagram of release schedule from ribociclib-induced G1/S arrest synchronization. (E-F) Representative histograms of cell cycle analysis of A549 (B) and LN308 (C) cancer cell lines treated with ribociclib for 1 day followed by ribociclib withdrawal for 0–3 days (D0-D3). Percentages of cells at different stages of the cell cycle are listed. (TIF) [file pone.0223555.s004.tif]

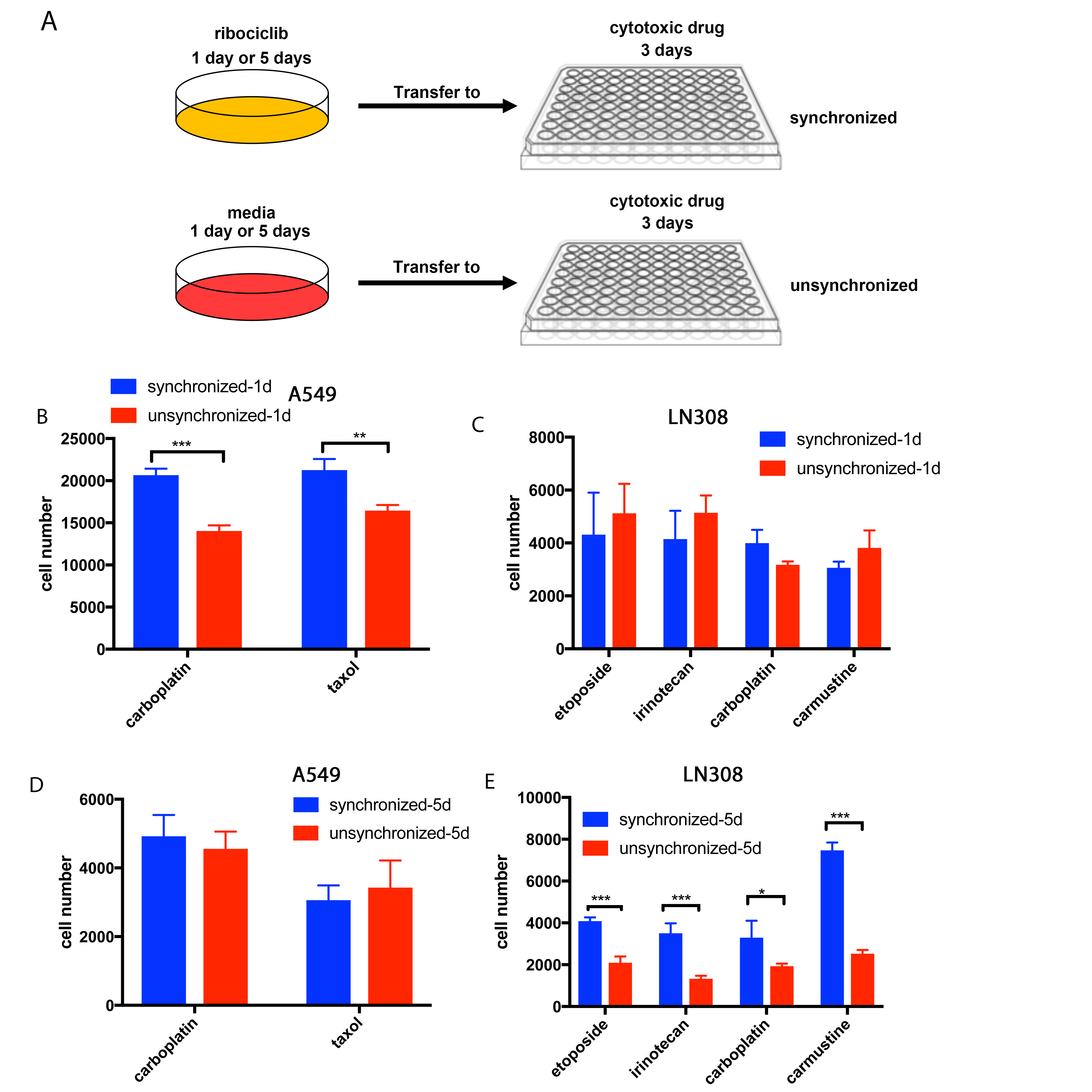

Supplement: S5 Fig — (A) Diagrams of experimental and control treatment schedule based on the synchronization-release schedules shown in Fig 3. (B-C) Representative graphs of 3 independent repeats of the cytotoxicity assay in indicated cells treated with indicated cytotoxic drugs after the 1-day synchronization-1-day release regime as shown in A. (D-E) Representative graphs of 3 independent repeats of the cytotoxicity assay in indicated cells treated with indicated cytotoxic drugs after the 5-day synchronization-1-day release regime as shown in A. All values are numbers of live cells remaining in culture at the end of treatment and presented as Mean (SD). P-value was calculated using 2-sided T-test: *, p<0.05; **, p <0.01; ***, p < 0.001. (TIF) [file pone.0223555.s005.tif]
