# Supplementary material for: Combining CDK4/6 inhibitors ribociclib and palbociclib with cytotoxic agents does not enhance cytotoxicity
Source: PLoS One. 2019 Oct 10;14(10):e0223555. doi: 10.1371/journal.pone.0223555 (PMC6786609; doi:10.1371/journal.pone.0223555)
Supplement: Attachment — Submitted filename: responses to review comments.pdf [file pone.0223555.s006.pdf]

Responses to Reviewers' comments. Reviewers' comments are in black font; our responses in blue.

Reviewer #1

Major comments:

1. Before driving the conclusion that the CDKI4/6 inhibitors should not be combined with anticancer drugs in clinical settings, other mechanisms of possible synergism must be considered. Authors do few attempts to address the issue in the discussion, 472-489, nevertheless, the hypothesis on ABC transporters (lines 477-480) is wrong. There is no need to consider ABC efflux transporters to represent a mechanism for antagonism in this case! The authors correctly mention that several previous studies revealed first/second generation of CDKI as potent inhibitors of ABC efflux transporters, which are commonly expressed in cancer cells (citing references 41-43). Nevertheless, they completely ignore the content of these articles, e.g. the recent study of Sorf et al., 2018 clearly show the synergistic cytotoxic and proapoptotic effect of combination of ribociclib with mitoxantrone and daunorubicin in ABC-expressing cells. This factor must be discussed, even though, it did not play probably role in the results of this study since the cell lines used (such as MDA-MB-231) are known for only negligible and non-physiological levels of ABC transporters. So it is very probably, that in the clinical conditions this effect of CDKI on ABC transporter-mediated efflux of cytotoxic drugs that results in synergism in cytotoxicity might overwhelm the antagonism described here. This must be considered and at least discussed properly!

We have re-phrased this section of the discussion to add other potential mechanisms and for better clarification of the ABC transporter connection (Lines 492-503).

2. Introduction lines 97-99 the authors mention „general dependency of many cytotoxic drugs on cell division ". This statement is only partly true, since e.g. alkylating agents, such as cisplatin are able to act also on quescent cells in G0 Phase. In your study you use several cytotoxic drugs, however their cell cycle phase specificity (or unspecificity) must be considered and the experimental results discussed in this context!

We change the statement "general dependency of many cytotoxic drugs on cell division" (lines 96-98) to "This observation was in part expected due to the positive correlation between cytotoxicity of many cytotoxic drugs and the rate of cell division."

We change the statement "The different dependencies on cell cycle phases for cytotoxic drugs are discussed" (lines 465-476) to "The six cytotoxic agents used in this study have distinct mechanisms of action. Although alkylating agents (e.g. carmustine, temozolomide in this study) and platinum-based agents (e.g. carboplatin) are not cell cycle dependent, fast dividing cells in general are more sensitive to these agents than

are non-dividing cells, suggesting that the cytotoxicity of these drugs are dependent on cell cycling. On the other hand, cell cycle dependent cytotoxic agents exert their cytotoxicity by disrupting cell cycle phase specific functions, thereby forcing associated cell cycle phase checkpoints, e.g. etoposide and irinotecan, S phase-specific topoisomerase inhibitors specifically target DNA replication and the S phase checkpoint, and paclitaxel, a microtubule-stabilizing agent specifically targets the M phase checkpoint. Despite these differences in their mechanisms of action, …"

3.The statistical analysis is completely missing in the methodological part – this must be filled properly. Re-consider also the statistical method used in evaluation of the data shown in Fig. 1 and 2. When comparing for example the effect of added ribociclib on the cisplatin alone the effect of sole ribociclib must be considered and thereby ANOVA should be better used.

We added the method description for statistical analysis used in this manuscript (Line 157-161) and also re-analyzed p-values by ANOVA for Figures 1, 2, 5, S1, S2, S3. Comparison between different treatments and ribociclib alone was also added in the graphs where appropriate.

Minor comments:

Page 17 lines 222-223 „…breast and lung and breast cancer cells "(„breast" is doubled here)

Corrected in line 226

Fig 1 A consistent units should be used in describing the concentration of applied drugs (preferring molarity)

A new IC50 table has replaced the previous version with consistent molarity concentration units (Figure 1A).

Reviewer #2

Major comments:

1. Conflicting results regarding the benefit of combining small molecule CDK4/6 inhibitors with standard cytotoxic chemotherapy have been reported. Results presented in this study may not be a general phenomenon for all of the small molecule CDK4/6 inhibitors and all of the cytotoxic agents. Therefore, the title should at least indicate the specific CDK4/6 inhibitor, ribociclib, used in this study.

Although we did not test all of the available CDK4/6 inhibitors, we did test 2 CDK4/6 inhibitors, ribociclib in all the experiments shown and palbociclib in two cell lines LN428 and A549 (Figure S2). The results for palbociclib are consistent with those for ribociclib. Therefore, we have changed to title to specify ribociclib and palbociclib.

2. While the results are interesting and important, however, they were conducted only in cell culture model. As there are major differences between cell culture model and preclinical animal models, it would be helpful if the authors could validate the results from cell culture study by conducting at least one preclinical animal study to demonstrate the major point of the research.

We appreciate the suggestion and agree that this is an important next step. We did acknowledge the limitation of our study due to its in vitro nature. However, conducting a preclinical animal model study properly will require both time and resources to first optimize pharmacokinetics and pharmacodynamics profiles of individual drugs and then in combinations in a breast cancer model before definitive experiments can proceed. This study is currently in planning, we anticipate at least 1-2 more years to complete. However, as we have pointed out in the manuscript, due to the nature of our observations with direct clinical implications regarding patient safety, we feel that it is important to publish the data in its current form to alert the field that extra precautions may be warranted when these two classes of drugs are combined in the clinic until more definitive preclinical confirmation can be obtained.
